# Transcranial Direct Current Stimulation on Different Targets to Modulate Cortical Activity and Dual-Task Walking in Individuals With Parkinson’s Disease: A Double Blinded Randomized Controlled Trial

**DOI:** 10.3389/fnagi.2022.807151

**Published:** 2022-02-07

**Authors:** Pei-Ling Wong, Yea-Ru Yang, Shih-Fong Huang, Jong-Ling Fuh, Han-Lin Chiang, Ray-Yau Wang

**Affiliations:** ^1^Department of Physical Therapy and Assistive Technology, National Yang Ming Chiao Tung University, Taipei, Taiwan; ^2^Division of Nerve Repair, Department of Neurosurgery, Taipei Veterans General Hospital, Taipei, Taiwan; ^3^Division of General Neurology, Department of Neurology, Neurological Institute, Taipei Veterans General Hospital, Taipei, Taiwan

**Keywords:** tDCS, different targets, single-session effects, dual-task gait, cortical activity, Parkinson’s disease

## Abstract

**Background:**

Transcranial direct current stimulation (tDCS) is a non-invasive brain stimulation to modulate cortical activity for improving motor function. However, the information of tDCS stimulation on different brain regions for dual-task walking and cortical modulation in Parkinson’s disease (PD) has not yet been compared.

**Objective:**

The objective of this study was to investigate the effects of different tDCS targets on dual-task gait performance and cortical activity in patients with PD.

**Methods:**

A total of 36 participants were randomly assigned to primary motor cortex (M1) tDCS, dorsal lateral prefrontal cortex (DLPFC) tDCS, cerebellum tDCS, or Sham tDCS group. Each group received 20 min of tDCS stimulation, except for the Sham group. Gait performance was measured by the GAITRite system during dual-task walking and single walking. Corticomotor activity of the tibialis anterior (TA) was measured using transcranial magnetic stimulation (TMS). The functional mobility was assessed using the timed up and go (TUG) test.

**Results:**

All participants showed no significant differences in baseline data. Following the one session of tDCS intervention, M1 (*p* = 0.048), DLPFC (*p* < 0.001), and cerebellum (*p* = 0.001) tDCS groups demonstrated significant improvements in dual-task gait speed compared with a pretest. The time × group interaction [*F*(3, 32) = 5.125, *p* = 0.005] was detected in dual-task walking speed. The *post hoc* Tukey’s test showed that the differences in gait speed were between the Sham tDCS group and the DLPFC tDCS group (*p* = 0.03). Moreover, DLPFC tDCS also increased the silent period (SP) more than M1 tDCS (*p* = 0.006) and Sham tDCS (*p* = 0.002).

**Conclusion:**

The results indicate that DLPFC tDCS exerted the most beneficial effects on dual-task walking and cortical modulation in participants with PD.

**Clinical trial registration:**

[http://www.thaiclinicaltrials.org/show/TCTR20200909005], Thai Clinical Trials Registry [TCTR20200909005].

## Introduction

Parkinson’s disease (PD) is a degenerative neurological disease due to the loss of dopaminergic neurons in the substantia nigra pars compacta in basal ganglia (BG) (Lees et al., 2009). With impaired interactions among cortico-BG-cerebellar circuits, the deficits in gait performance are frequently seen in individuals with PD (Hausdorff et al., 1998). In addition to classical motor symptoms, cognitive symptoms are widely accepted as part of the clinical feature in individuals with PD. These motor and cognitive impairments increased their difficulties to perform complex daily activities, such as dual-task walking (i.e., responding to a cognitive demanding task while walking) (Benecke et al., 1986; Raffegeau et al., 2019). According to a meta-analysis, the gait speed and stride length decreased under the condition of dual-task walking as compared with single walking in people with PD (Raffegeau et al., 2019). These significant difficulties in dual-task walking may lead to increased disability, fall risks, and decreased quality of life in people with PD (Kelly et al., 2012). Therefore, how to improve the dual-task walking performance is crucial for people with PD.

In addition to deficits in cortico-BG-cerebellar circuits, 33 studies demonstrated abnormal activity in primary motor cortex (M1), dorsal lateral prefrontal cortex (DLPFC), and cerebellum during dual-task performance in patients with PD (Wu and Hallett, 2005; Nieuwhof et al., 2016; Al-Yahya et al., 2019). It has been suggested that abnormal plasticity within M1 reflects a loss of coordination among the BG, cerebellar, and cortical inputs and eventually causes motor impairments in PD (Gaspar et al., 1991; Kishore et al., 2014). The decreased M1 inhibition during resting was reported in people with PD by transcranial magnetic stimulation (TMS) (Rossini et al., 2015), which may be one of the compensations for the cortico-BG-cerebellar deficit. Furthermore, the pattern of hypo-activation between the cortical area and striatum was associated with gait impairment in PD (Shine et al., 2013). Therefore, modulating brain activities might be a strategy for motor improvement, especially the complex movement, such as dual-task walking in individuals with PD.

The transcranial direct current stimulation (tDCS) is a non-invasive technique to modulate cortical excitability (Sánchez-Kuhn et al., 2017). Anodal tDCS has been considered not only to alter cortical excitability but also to exert subcortical effects (Polanía et al., 2012). Fregni et al. (2006) indicated that a single session of M1 tDCS improved upper extremity performance. Moreover, Ferrucci et al. (2016) demonstrated that 5 sessions of cerebellum tDCS decreased the disease severity as indicated by the Unified Parkinson’s Disease Rating Scale (UPDRS). The previous study suggested that the lateral cerebellar region plays an important role in the complex motor task, such as dual-task walking (Ilg and Timmann, 2013). Furthermore, a recent review suggested that cerebellum may be the potential tDCS target area to improve the gait performance in people with PD (Potvin-Desrochers and Paquette, 2021). In contrast, as mentioned earlier, walking in daily activities demands interactions between motor and cognitive control, particularly, executive function (Yuan and Raz, 2014). The DLPFC has been recognized as the key area for executive function, and the relative DLPFC activations during gait can be demonstrated *via* dual-task walking (Collette et al., 2005; Beurskens et al., 2014). Evidence also showed that a single session of DLPFC tDCS improved balance and mobility (Manenti et al., 2014; Lattari et al., 2017). Taking together, the potential use of tDCS has been demonstrated for people with PD in neurorehabilitation. However, it is not known whether different tDCS targets would modulate the brain differently to result in different effects on motor performance. Therefore, this study aimed to compare the different tDCS targets in brain modulation and dual-task walking performance in individuals with PD.

## Materials and Methods

### Subjects

**FIGURE 1:**
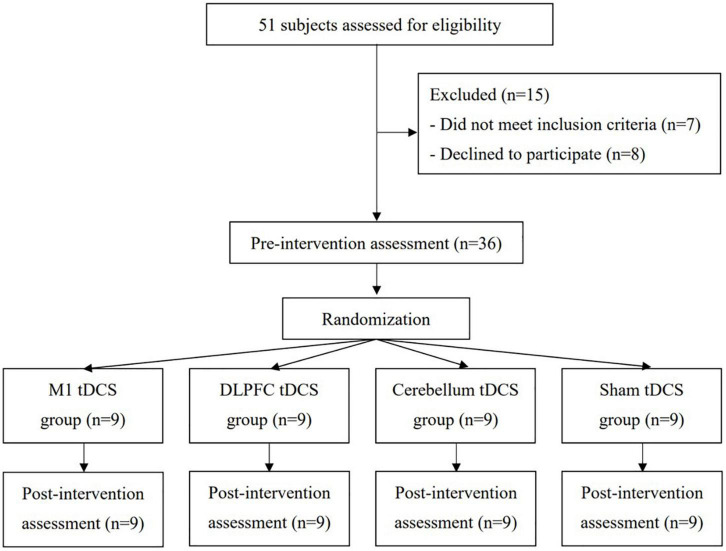
Flowchart of the patient inclusion and study procedures.

This study protocol was approved by the Institutional Review Board of Taipei Veterans General Hospital and Ministry of Health and Welfare. This trial was registered at https://www.clinicaltrials.in.th/(TCTR20200909005) and conformed to the CONSORT checklist. Participants who were diagnosed with idiopathic PD by neurologists (J-LF and H-LC) were recruited from Taipei Veterans General Hospital. The age, gender, duration since the diagnosis of PD, and medications were obtained from the detailed clinical interviews and medical charts. Inclusion criteria were as follows: (1) stages 1–3 on the Hoehn and Yahr scale, (2) ability to walk independently for at least 10 m without the use of walking aids, (3) stable medical condition, and (4) a score of ≥ 24 on the Mini-Mental State Examination (MMSE). Exclusion criteria were as follows: (1) history of diseases or conditions known to interfere with participating in this study (e.g., epilepsy or metal implants in the brain) and (2) history of using central nervous system medications other than for PD, e.g., antiepileptic or antidepressant drugs in recent months. In total, 51 individuals were identified as potential subjects. Of these, 36 participants provided informed consent for participation in this study (Figure 1).

### Experimental Design

This study was a double-blinded, randomized, controlled trial with pre- and post-measurements. An individual who was not involved in this study selected the sealed envelopes to assign participants to one of the four groups (i.e., M1 tDCS group, DLPFC tDCS group, cerebellum tDCS group, and Sham tDCS group) by the block of 2 randomizations. The cortical activities followed by gait performance were measured before (pretest) the real (or Sham) tDCS by the assessor who was blinded to the group assignment (assessor blinded) (Figure 1). Participants were blinded to their group assignment (participants blinded) and received one session of real or Sham tDCS for 20 min according to the group assignment. After 20 min of tDCS, all participants were measured the cortical activities immediately after tDCS and gait performance for 30 min after tDCS. All interventions and assessments were carried out with patients in the “on” status.

### Intervention

The stimulation was delivered by a current stimulator (Eldith DC Stimulator, NeuroConn, Germany) through a pair of 35 cm^2^ electrodes with a maximal output of 2 mA. The stimulation intensity was set to 2 mA for 20 min.

a. M1 tDCS group: The anode was placed over the M1 of the dominant hemisphere (C3 according to EEG 10/20 system), and the cathode was placed over the contralateral supraorbital ridge (Fregni et al., 2006).
b. DLPFC tDCS group: The anode was placed over the DLPFC of the dominant hemisphere (F3 according to EEG 10/20 system), and the cathode was placed over the contralateral supraorbital ridge (Fregni et al., 2006; Lattari et al., 2017).
c. Cerebellum tDCS group: The anode was placed 1 cm below and 2 cm lateral to the inion over the dominant hemisphere, and the cathode was placed over the contralateral supraorbital ridge (Ferrucci et al., 2015).
d. Sham tDCS group: The electrodes were positioned as described in the M1 tDCS group. However, the current was delivered only for the first 60 s, with a ramp up and ramp down for 30 s.

### Outcome Measures

#### Primary Outcome Measures

The primary outcome of this study was dual-task walking performance measured by a GAITRite system (CIR system, Inc., Havertown, PA, United States) (Yang et al., 2019). The GAITRite system is 4.75 m long and 0.9 m wide, and the pressure-sensitive area of the walkway is 4.30 m long and 0.61 m wide. The dual-task walking was walking while performing serial subtracting by three, starting from a randomized 3-digit number at a comfortable speed. The walking trial was repeated three times with 60 s rest in between. The average of the three trials of each walking condition was used for data analysis. Gait parameters of interest were speed, cadence, stride time, stride length, and coefficients of variation (CV) of stride time and stride length. The formula of CV is standard deviation/mean × 100%. A lower CV value means a more consistent gait pattern (Yang et al., 2013). In addition, the dual-task cost (DTC) was calculated to indicate the dual-task interference. The formula of DTC was DTC = (dual-task walking speed – single-task walking speed)/single-task walking speed × 100% (Yang et al., 2019).

#### Secondary Outcome Measures

The secondary outcomes included corticomotor activity, single walking performance, and functional mobility.

##### Corticomotor Activity

The resting motor threshold (RMT), motor evoked potentials (MEPs), and silent period (SP) duration of the tibialis anterior (TA) elicited by TMS (Magstim 200 magnetic stimulator; Magstim Company, Whiteland, Dyfed, United Kingdom) were used to indicate the corticomotor activity. The MEPs of TA were recorded by an electromyographic (EMG) machine in response to TMS delivered through a double-cone coil placed on the M1 with participants lying supine wearing a fitted cap marked with a coordinate system (distance, 1 cm). The optimal scalp location (hot spot) was determined by moving the TMS stimulator over the scalp in 1-cm steps. Once the hot spot was identified, a single-pulse TMS was delivered to the location to determine the RMT, as the lowest stimulus intensity necessary to elicit MEPs greater than 0.05-mV peak-to-peak amplitude in at least 5 of 10 consecutive stimuli (Yang et al., 2013). The RMT was expressed as a percentage of maximum stimulator output, which reflects the excitability of motor cortex (Groppa et al., 2012). The MEPs were measured at an intensity of 120% RMT, and the peak-to-peak amplitudes of the MEPs of 10 trials were collected and averaged. The amplitude of MEPs is thought to represent the corticospinal excitability of the M1 (Groppa et al., 2012). Both RMT and MEP were considered to be mediated by the glutamatergic system indicated by the TMS-pharmacological study (Kapogiannis and Wassermann, 2008; Paulus et al., 2008). The SP duration was determined during isometric voluntary contraction of TA. Ten magnetic stimuli were applied at an intensity of 120% RMT, while the participant performed maximum of 20% voluntary contraction. The intensity used in the post-assessment was the same as that used in the pre-assessment. The SP duration was determined from the MEP onset to the recurrence of at least 50% of EMG background activity (Yang et al., 2013). The neurophysiological phenomenon of SP is thought to be due to inhibition mechanisms of the motor cortex mediated through the GABAergic system (Werhahn et al., 1999).

##### Single Walking Performance

The single walking performance was also measured using the GAITRite system. For a single walking performance, the participants walked at their comfortable speed without additional tasking. The average of the three trials was used for data analysis.

##### Timed Up and Go Test

The timed up and go (TUG) was used to evaluate the functional mobility. The participants were seated in a chair and were instructed to stand up, walk 3 m, turn around, walk back to the chair, and then sit down. We recorded the time needed to complete this task. A high reliability of this test has been documented in individuals with PD (Morris et al., 2001).

### Statistical Analysis

All analyses were performed using the SPSS version 24.0. Descriptive statistics [mean ± standard deviation, frequency, or median (interquartile range)] were generated for all variables. The Shapiro–Wilk test was used to assess the normal distributions. The intergroup difference of baseline (pretest) data was analyzed by using the Kruskal–Wallis test and one-way ANOVA for continuous variables or χ^2^ test for nominal scales. Accordingly, the two-way repeated measures ANOVA (group × time) was used for intergroup comparisons of dual-task walking, single walking, and TUG performance, followed by the *post hoc* Tukey’s test with the Bonferroni correction, which multiplied the uncorrected *p*-values by 6 for multiple comparisons between four groups, if there was group × time effect. The *post hoc* paired *t*-test was used to examine significance between pre- and post-data if there was a significant time effect. The Kruskal–Wallis one-way ANOVA was used for intergroup comparisons of change values in corticomotor activity due to not being normally distributed, followed by the *post hoc* Mann–Whitney *U* tests with the Bonferroni correction. The intragroup difference was thus analyzed by the Wilcoxon signed-rank test. The change values of corticomotor activity were calculated by subtracting the baseline data from the post-intervention data. Statistical significance was set at *p* < 0.05. The sample size was calculated using the G-Power version 3.1.9.7. Although the effect size was 0.85 for tDCS in improving dual-task gait performance in patients with PD (Mishra and Thrasher, 2021), in this study, we chose a relatively smaller effect size of 0.518 according to the study by Kaski et al. (2014). The total sample size was required to be 36 (9 per group) with a power of 0.80 and a two-tailed alpha level of 0.05 to detect a difference in gait performance.

## Results

A total of 51 patients were screened for the eligibility of participating in this study. As a result, 36 patients were included in this study and were randomly assigned to the M1 tDCS group (*n* = 9), DLPFC tDCS group (*n* = 9), cerebellum tDCS group (*n* = 9), or Sham tDCS group (*n* = 9). Participants received 20 min of tDCS according to their group assignment. None of them reported any adverse events or withdrew from this study (Figure 1). No significant differences between groups were found in baseline demographic characteristics (Table 1) and all outcome measures at the pre-intervention assessment.

**TABLE 1:** Demographic characteristics of included participants with Parkinson’s disease.

Group	M1 group (*n* = 9)	DLPFC group (*n* = 9)	Cerebellum group (*n* = 9)	Sham group (*n* = 9)	*P* value
Age (years)	54.20 ± 4.1	50.09 ± 2.4	61.30 ± 7.9	58.30 ± 8.0	0.60
Gender (M/F)	8/1	6/3	2/7	3/6	<0.01
H&Y stage	1.89 ± 0.6	1.67 ± 0.5	2.13 ± 0.6	1.78 ± 0.7	0.75
More affected side (L/R)	2/7	1/8	2/7	1/8	0.85
Duration of diagnosed (months)	93.54 ± 68.2	73.81 ± 39.2	49.11 ± 39.3	100.18 ± 147.0	0.43
UPDRS	33.22 ± 13.1	25.56 ± 17.0	24.22 ± 9.9	23.44 ± 14.7	0.48
MMSE	28.11 ± 1.8	28.89 ± 1.8	27.33 ± 2.2	28.89 ± 2.0	0.29
LEDD (mg)	592.1 ± 208.2	603.89 ± 357.3	468.22 ± 212.1	426.11 ± 243.7	0.41

*M, male; F, female; H&Y, Hoehn and Yahr stage; L, left; R, right; MMSE, Mini-Mental State Examination; LEDD: levodopa equivalent daily dosage.*

### Dual-Task Walking Performance

**FIGURE 2:**
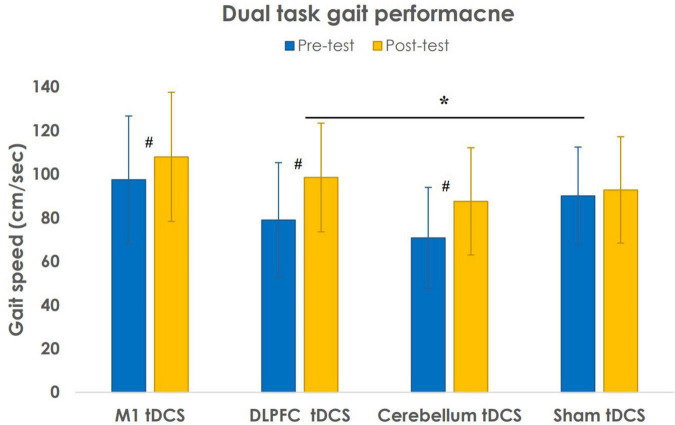
Results of gait speed during dual-task walking performance after different transcranial direct current stimulation (tDCS) stimulations. Data are presented as the mean ± SD. **^#^***P* < 0.05: intragroup comparison. *P < 0.05: intergroup comparison.

Table 2 shows the dual-task walking performance at pre- and post-intervention for 4 study groups. Regarding the dual-task gait parameters, there was no group effect [*F*(3, 32) = 2.237, *p* = 0.103] but a significant effect of time [*F*(3, 32) = 56.616, *p* < 0.001] and time × group interaction [*F*(3, 32) = 5.125, *p* = 0.005]. The *post hoc* Tukey’s test with the Bonferroni correction showed that the differences in gait speed were between the Sham tDCS group and the DLPFC tDCS group (*p* = 0.03) (Figure 2). The cadence showed no group effect [*F*(3, 32) = 1.723, *p* = 0.182) but a significant effect of time [*F*(3, 32) = 41.497, *p* < 0.001] and time × group interaction [*F*(3, 32) = 5.180, *p* = 0.005]. However, the *post hoc* Tukey’s test with the Bonferroni correction did not show any group difference in cadence. The stride time showed no group effect [*F*(3, 32) = 1.487, *p* = 0.237] but a significant effect of time [*F*(3, 32) = 9.628, *p* = 0.004) and time × group interaction [*F*(3, 32) = 3.649, *p* = 0.023]. However, the *post hoc* Tukey’s test with the Bonferroni correction did not show any group difference in stride time. The stride length showed no group effect [*F*(3, 32) = 2.267, *p* = 0.100] but a significant effect of time [*F*(3, 32) = 22.069, *p* < 0.001] and time × group interaction [*F*(3, 32) = 3.340, *p* = 0.031]. However, the *post hoc* Tukey’s test with the Bonferroni correction did not show any group difference in stride length.

**TABLE 2:** Dual task walking performance after different tDCS interventions.

Group	M1 group (*n* = 9)	DLPFC group (*n* = 9)	Cerebellum group (*n* = 9)	Sham group (*n* = 9)	Time	Group	Time × group	*Post-hoc^a^*
		
					*p*-value,	*p*-value,	*p*-value,	
					*F*-value	*F*-value	*F*-value, η^2^	
Speed_(cm/sec)_					<0.001,	0.103,	0.005,	DLPFC vs. Sham
					*F*(3,32) = 56.616	*F*(3,32) = 2.237	*F*(3,32) = 5.125, 0.325	
Pre	97.48 ± 29.2	78.99 ± 26.3	70.74 ± 23.2	90.01 ± 22.4				
Post	107.94 ± 29.6**^#^**	98.49 ± 24.9**^#^**	87.54 ± 24.6**^#^**	92.77 ± 24.4				
Cadence _(step/min)_					<0.001,	0.182,	0.005,	–
					*F*(3,32) = 41.497	*F*(3,32) = 1.723	*F*(3,32) = 5.180, 0.327	
Pre	108.60 ± 12.9	104.87 ± 20.1	87.24 ± 27.9	108.41 ± 19.2				
Post	113.32 ± 14.2	119.18 ± 16.8**^#^**	93.33 ± 19.2**^#^**	111.20 ± 20.5				
ST _(sec)_					0.004,	0.237,	0.023,	–
					*F*(3,32) = 9.628	*F*(3,32) = 1.487	*F*(3,32) = 3.649, 0.255	
Pre	1.12 ± 0.1	1.19 ± 0.2	1.23 ± 1.0	1.16 ± 0.3				
Post	1.08 ± 0.1	1.02 ± 0.1**^#^**	1.15 ± 0.3	1.14 ± 0.3				
SL _(cm)_					<0.001,	0.100,	0.031,	–
					*F*(3,32) = 22.069	*F*(3,32) = 2.267	*F*(3,32) = 3.340, 0.238	
Pre	106.12 ± 22.5	89.02 ± 18.9	84.83 ± 24.6	100.11 ± 18.3				
Post	112.95 ± 22.0**^#^**	98.32 ± 14.7**^#^**	92.18 ± 18.8**^#^**	99.30 ± 18.7				
ST variability _(%)_					0.047,	0.832,	0.239,	–
					*F*(3,32) = 4.287	*F*(3,32) = 0.291	*F*(3,32) = 1.476, 0.122	
Pre	5.63 ± 5.5	5.06 ± 4.5	16.23 ± 18.7	4.80 ± 3.1				
Post	3.59 ± 2.5	3.13 ± 1.1	7.34 ± 3.8	4.58 ± 4.1				
SL variability _(%)_					0.385,	0.319,	0.936,	–
					*F*(3,32) = 0.777	*F*(3,32) = 1.217	*F*(3,32) = 0.138, 0.013	
Pre	4.64 ± 3.3	5.20 ± 3.0	7.16 ± 4.1	4.38 ± 2.1				
Post	4.11 ± 3.3	4.15 ± 2.0	6.39 ± 3.3	4.46 ± 2.2				
DTC _(%)_					0.006,	0.078,	0.140,	–
					*F*(3,32) = 8.779	*F*(3,32) = 2.493	*F*(3,32) = 1.957, 0.155	
Pre	−15.19 ± 17.5	−20.31 ± 25.7	−18.31 ± 22.1	−10.14 ± 11.2				
Post	−12.27 ± 9.0	−13.53 ± 18.4^#^	−13.54 ± 20.0	−8.97 ± 14.0				

*Data are presented as the mean ± SD (The Shapiro–Wilk test was used to determine the values are normally distributed). ST, stride time; SL, stride length; DTC, dual task cost. ^#^p < 0.05 for intragroup comparison (Analyzed using paired t-test). ^a^The Tukey’s post-hoc test with Bonferroni correction was used to determine the intergroup differences.*

Furthermore, two-way repeated ANOVA indicated several significant time effects. The *post hoc* paired *t*-test showed a significant increase in gait speed [*t*(8) = −6.963, *p* < 0.001], cadence [*t*(8) = −6.659, *p* < 0.001], and stride length [*t*(8) = −3.761, *p* = 0.006] and a decrease in stride time [*t*(8) = 4.600, *p* = 0.002] after DLPFC tDCS intervention. In addition, the *post hoc* paired *t*-tests indicated that patients in cerebellum tDCS group significantly increased in gait speed [*t*(8) = −5.231, *p* = 0.001], cadence [*t*(8) = −3.499, *p* = 0.008], and stride length [*t*(8) = −2.610, *p* = 0.031] and patients in M1 tDCS group significantly increased in gait speed [*t*(8) = −2.338, *p* = 0.048] and stride length [*t*(8) = −2.492, *p* = 0.037] after tDCS intervention.

### Corticomotor Activity

**FIGURE 3:**
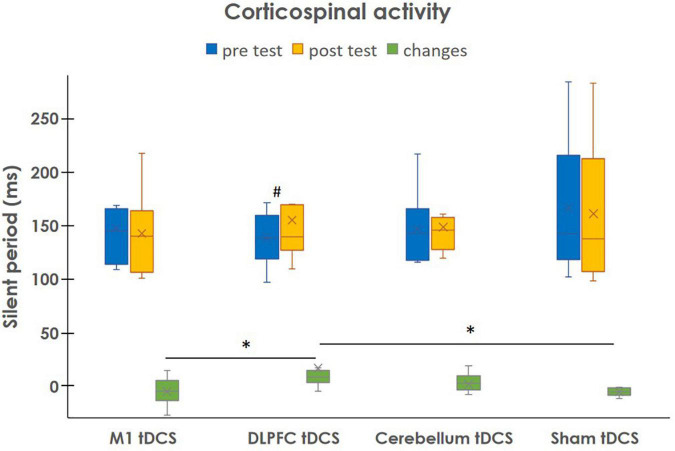
Changes in silent period of ipsilateral hemisphere relatively to the stimulating side after different tDCS stimulations. Data are presented as the median (interquartile range). ^#^*P* < 0.05: intragroup comparison. *P < 0.05: intergroup comparison.

Table 3 shows the cortical activity of M1 measured by the TMS before and after tDCS interventions. After the DLPFC tDCS stimulation, the SP of stimulating hemisphere increased significantly more than M1 tDCS (*p* = 0.038) and Sham tDCS (*p* = 0.001) (Figure 3). However, there was no significant difference in other groups. In contrast, the corticomotor activity of non-stimulating hemisphere did not change in this study.

**TABLE 3:** Corticospinal activity after different tDCS interventions.

Group	M1 group (*n* = 9)		DLPFC group (*n* = 9)		Cerebellum group (*n* = 9)		Sham group (*n* = 9)		*p*
	Pre	Post	Pre	Post	Pre	Post	Pre	Post	
RMT_IH_ (%)	51.00	55.00	58.00	58.00	65.00	65.00	61.80	62.00	
	(46.00, 58.00)	(50.00, 62.00)	(47.00, 67.00)	(47.00, 70.00)	(60.00, 69.00)	(59.00, 69.00)	(53.50, 73.50)	(53.00, 74.00)	
Change values	2.00 (0.00, 2.00)		0.00 (−1.00, 1.00)		0.00 (−1.00, 0.00)		0.00 (−1.00, 0.00)		0.063
RMT_CH_ (%)	53.00	55.00	56.00	58.00	59.00	60.00	52.00	52.00	
	(49.00, 57.00)	(48.00, 61.0 0)	(49.50, 75.00)	(50.00, 72.00)	(48.00, 63.00)	(53.00, 62.00)	(45.00, 62.00)	(45.00, 62.00)	
Change values	2.00 (−1.00, 3.00)		0.00 (−2.50, 1.50)		2.00 (−1.00, 5.00)				0.312
MEP_IH_ (uV)	418.66	341.38	416.77	315.18	630.78	763.50	329.06	292.61	
	(289.89, 593.17)	(204.45, 773.70)	(276.68, 610.96)	(395.45, 208.47)	(547.20, 858.11)	(361.21, 1068.26)	(159.31, 483.07)	(138.75, 387.26)	
Change values	30.79 (−164.05, 367.42)		−71.10 (−285.29, −41.78)		69.07 (−76.46, 248.79)		−3.01 (−69.25, 8.40)		0.240
MEP_CH_ (uV)	533.55	696.96	408.38	491.19	589.37	516.62	415.82	431.05	
	(315.18, 939.84)	(289.53, 967.77)	(252.60, 580.35)	(333.53, 773.04)	(494.40, 778.99)	(296.92, 750.30)	(247.63, 601.53)	(220.53, 556.67)	
Change values	47.14 (−344.16, 348.62)		67.87 (−59.22, 224.94)		−103.92 (−367.59, 177.84)		−20.77 (−43.49, 8.17)		0.678
SP_IH_ (ms)	144.59	140.02	138.21	139.33	142.29	145.58	141.98	137.44	
	(113.82, 165.44)	(105.91, 163.32)	(118.58, 159.36)	(126.41, 169.31)^#^	(117.51, 165.21)	(127.09, 157.54)	(118.17, 215.04)	(106.77, 211.84)	
Change values	−5.11 (−13.52, 5.19)		8.50 (3.13, 14.79)^a,s^		2.48 (−3.36, 9.72)		−5.25 (−8.67, −1.50)		0.007
SP_CH_ (ms)	134.68	132.57	135.59	135.18	136.99	142.94	127.95	135.55	
	(124.78, 151.61)	(118.94, 160.67)	(116.16, 151.57)	(117.18, 148.02)	(123.75, 257.60)	(126.98, 155.93)	(118.98, 141.38)	(121.44, 145.47)	
Change values	2.25 (−14.66, 9.06)		−0.41 (−2.84, 1.85)		0.83 (−3.69, 6.39)		1.84 (−6.51, 13.81)		0.822

*Data are presented as the median (Interquartile range) (The Shapiro–Wilk test was used to determine the values are not normally distributed). IH, ipsilateral hemisphere relatively to the stimulating side; CH, contralateral hemisphere relatively to the stimulating side. Change values were calculated by subtracting the baseline data from the post-test data. ^a^p < 0.05 as compared with M1 group; ^s^p < 0.05 as compared with Sham group.*

### Single Walking Performance

Table 4 shows the single walking performance after different tDCS interventions. We found no significant time and group interaction for all gait parameters of single walking but a significant time effect. The *post hoc* paired *t*-test showed that a significant increase in gait speed [*t*(8) = −2.528, *p* = 0.035] and cadence [*t*(8) = −3.291, *p* = 0.011] after DLPFC tDCS intervention. In M1 tDCS group, the *post hoc* paired *t*-test showed a significant increase in stride length [*t*(8) = −3.315, *p* = 0.011] after tDCS intervention.

**TABLE 4:** Single walking and timed up and go performance after different tDCS interventions.

Group	M1 group (*n* = 9)	DLPFC group (*n* = 9)	Cerebellum group (*n* = 9)	Sham group (*n* = 9)	Time	Group	Time × group
					*p*-value, F-value	*p*-value, F-value	*p*-value, F-value, η^2^
Single walking							
Speed _(cm/sec)_					<0.001, *F*(3,32) = 15.272	0.056, *F*(3,32) = 2.800	0.197, *F*(3,32) = 1.653, 0.134
Pre	113.94 ± 22.1	101.14 ± 21.4	87.19 ± 21.8	101.17 ± 18.9			
Post	121.59 ± 26.6	114.30 ± 19.3^#^	92.18 ± 22.7	103.50 ± 19.4			
Cadence _(step/min)_					0.012, *F*(3,32) = 7.176	0.117, *F*(3,32) = 2.119	0.058, *F*(3,32) = 2.764, 0.206
Pre	120.09 ± 8.4	116.86 ± 12.0	107.78 ± 11.9	120.26 ± 8.4			
Post	121.41 ± 12.0	124.44 ± 12.5^#^	112.16 ± 13.3	119.93 ± 10.0			
ST _(sec)_					0.792, *F*(3,32) = 0.070	0.102, *F*(3,32) = 2.250	0.052, *F*(3,32) = 2.858, 0.211
Pre	1.01 ± 0.1	1.04 ± 0.1	1.12 ± 0.1	0.88 ± 0.3			
Post	1.01 ± 0.1	0.97 ± 0.1	1.09 ± 0.2	1.01 ± 0.1			
SL _(cm)_					<0.001, *F*(3,32) = 16.406	0.067, *F*(3,32) = 2.623	0.130, *F*(3,32) = 2.023, 0.159
Pre	113.55 ± 18.8	103.31 ± 14.9	96.41 ± 16.4	100.68 ± 14.6			
Post	120.33 ± 19.7^#^	110.27 ± 11.0	97.59 ± 17.7	102.92 ± 14.0			
ST variability _(%)_					0.049, *F*(3,32) = 4.190	0.298, *F*(3,32) = 1.278	0.589, *F*(3,32) = 0.650, 0.057
Pre	3.07 ± 1.4	3.08 ± 1.6	6.06 ± 5.5	6.07 ± 9.2			
Post	1.89 ± 0.9	2.90 ± 0.8	3.38 ± 1.2	2.58 ± 1.7			
SL variability _(%)_					0.134, *F*(3,32) = 2.369	0.743, *F*(3,32) = 0.415	0.496, *F*(3,32) = 0.813, 0.071
Pre	4.10 ± 2.9	3.86 ± 3.1	4.86 ± 3.2	3.68 ± 1.8			
Post	3.56 ± 2.8	4.25 ± 2.9	6.25 ± 3.4	2.53 ± 1.0			
Timed up and go _(sec)_					0.939, *F*(3,32) = 0.006	0.832, *F*(3,32) = 0.289	0.335, *F*(3,32) = 1.174, 0.099
Pre	9.59 ± 1.9	10.92 ± 2.1	13.56 ± 2.9	11.83 ± 4.0			
Post	9.92 ± 1.9	10.58 ± 1.4	13.26 ± 2.2	12.18 ± 3.3			

*Data are presented as the mean ± SD (the Shapiro–Wilk test was used to determine the values are normally distributed).*

*ST, stride time; SL, stride length.*

*^#^P < 0.05 for intragroup comparison (analyzed using paired t-test).*

### Timed Up and Go Performance

The results of TUG after different tDCS are shown in Table 4. We found no significant group effect [*F*(3, 32) = 0.289, *p* = 0.832], time effect [*F*(3, 32) = 0.006, *p* = 0.939], and group × time interactions [*F*(3, 32) = 1.174, *p* = 0.335]. In addition, there was no significant difference in intragroup comparisons.

## Discussion

This randomized, double-blinded, controlled trial was the first study to compare the immediate neuromodulation effects of different tDCS targets on dual-task walking performance in individuals with PD. In this study, we found that only the tDCS on DLPFC increased cortical inhibition and exerted the most beneficial effects to improve dual-task walking in people with PD as compared with tDCS on M1 or Sham tDCS.

In this study, the improvements in dual-task walking coupled with increased SP duration were demonstrated after DLPFC tDCS. The change in dual-task gait speed was highly correlated with change in SP (Spearman’s correlation ρ = 0.733, *p* = 0.025). Wu et al. (2007) noted that shorter SP duration was associated with worse PD symptoms. We previously found the lengthening in SP duration and improvement of single walking performance after the combination of high-frequency repetitive transcranial magnetic stimulation (rTMS) and treadmill training in patients with PD (Yang et al., 2013). Fisher et al. (2008) demonstrated the SP lengthening associated with walking improvements after treadmill training and thus speculated SP lengthening could restore the normal motor processing in people with PD. Recent studies have provided the evidence for the potential of one session of DLPFC tDCS to enhance dopamine release in the striatum (Fonteneau et al., 2018; Fukai et al., 2019). A previous study also reported that dopaminergic treatment could prolong SP duration in patients with PD and suggested that SP may be modulated by the dopamine system (Nakashima et al., 1995). Furthermore, the recent study showed tDCS-induced dopamine release and GABA changes, which contributes to the phenomenon of SP (Bunai et al., 2021). Taking together, the beneficial motor effects of DLPFC tDCS may be related to dopamine release, therefore modulating the cortical inhibition in individuals with PD.

In contrast, dual tasking exacerbates the gait impairments in people with PD, suggesting the overloaded recruitment of prefrontal cortex (cognitive overloaded) under dual-task walking (Strouwen et al., 2015). DLPFC has been recognized as the key area for executive function which involves in many daily activities, especially dual-task walking (Lu et al., 2015). Therefore, we speculated that the improvements in dual-task walking after DLPFC tDCS may also be resulted from direct modulation of DLPFC. In this study, the participants walked faster by 24% under cognitive dual tasking after one session of DLPFC tDCS intervention. Mishra and Thrasher (2021) also reported that the increase of dual-task gait speed after a single session of DLPFC tDCS was more than Sham tDCS in patients with PD. Similarly, tDCS targeting the DLPFC has been reported to improve dual-task gait performance in older adults and people with stroke (Zhou et al., 2021). In contrast, we previously noted that the dual-task gait training for 12 sessions resulted in a 20% improvement of cognitive dual-task walking speed in people with PD (Yang et al., 2019). Therefore, DLPFC tDCS is an effective intervention to immediately improve dual-task walking ability for individuals with PD.

However, it should be mentioned that the DLPFC tDCS group did not improve significantly more in single walking and TUG performance as compared with other groups, although the DLPFC tDCS group showed a pre-post significant improvement in single walking. This may indicate that the dual-task walking performance is more sensitive to reflect the response to intervention, and single walking and TUG performance may need cumulative tDCS interventions for a significant improvement. In addition, it is interesting to note that the RMT and MEP did not change significantly after DLPFC tDCS. Studies have reported that RMT is normal and MEP is variable in people with PD (Lefaucheur, 2005; Rossini et al., 2015). However, decreased SP has been reported consistently in patients with PD (Wu et al., 2007). Therefore, measurement of SP may be a better indicator to reflect cortical activity changes during disease progression and in response to treatment than RMT and MEP in individuals with PD.

Regarding the cerebellum tDCS, Workman et al. (2020) did not observe a significant single-session effect in single walking performance in people with PD. However, Jayaram et al. (2012) found that one session of anodal cerebellum tDCS resulted in better adaptation on a split-belt treadmill than Sham tDCS in healthy subjects. This study noted that the cerebellum tDCS exerted a significant within-group improvement in dual-task walking but not in single walking and TUG performance. However, such within-group improvement did not couple with significant changes in SP duration. This result lent us to speculate the vestibulocerebellum pathways, which are majorly involved in posture and balance control (Purves et al., 2001), may be modulated by cerebellum tDCS, but this warrants further exploration.

It also drew our attention that one session of anodal tDCS over M1 did not improve the walking performance, and such results were consistent with the results reported by Verheyden et al. (2013). Although Fregni et al. (2006) demonstrated that M1 tDCS improves UPDRS motor scores, limited improvement in the UPDRS gait-related items was noted. Moreover, Schabrun et al. (2016) demonstrated that M1 tDCS did not enhance the effect of dual-task gait training and suggested that M1 tDCS may not be an effective application to improve dual-task walking in individuals with PD. Considering the results of previous studies and this study, it still needs more investigations to establish the beneficial effects of tDCS over M1 on walking performance in people with PD.

Some limitations should be mentioned regarding this study. First, the relatively small number of participants in each group may lead to type II error. The limited sample size and heterogeneity of patients with PD must be considered when generalizing the study results. Second, the included participants were with mild to moderate disease severity (Hoehn and Yahr stages I–III), and the outcomes were measured only at “on” status. Therefore, our findings may only be applicable to individuals with mild to moderate PD at “on” status. Third, we only investigated the post-intervention effects of single-session tDCS, but the maintenance effects or accumulative effects of tDCS are not known. Fourth, we applied 2 mA for all targets because the current with 2 mA was most commonly used in the previous studies, especially in the studies focusing on walking ability (Ferrucci et al., 2015; Liu et al., 2021). However, the best parameter for tDCS in different targets may be different. Therefore, the results may not represent the best effect of tDCS in these targets. Further studies may need to establish the best stimulating intensity of tDCS in various targets. Finally, it has been noted that a single session of DLPFC tDCS could improve cognitive function according to the results of a meta-analysis (Dedoncker et al., 2016). However, we did not measure cognitive performances in this study. Therefore, the cognitive improvement cannot be excluded from the beneficial effects of DLPFC tDCS.

## Conclusion

The results suggest that one session of DLPFC tDCS can be recommended to improve dual-task walking. Further research is needed to explore the effects of multisessions of DLPFC tDCS.

## Data Availability Statement

The raw data supporting the conclusions of this article will be made available by the authors, without undue reservation.

## Ethics Statement

The studies involving human participants were reviewed and approved by Institutional Review Board of Taipei Veterans General Hospital. The patients/participants provided their written informed consent to participate in this study.

## Author Contributions

P-LW and R-YW conceived and designed the experiments. P-LW performed the experiments. P-LW and Y-RY analyzed the data. J-LF and H-LC confirmed the medical diagnosis of subjects and recruited subjects. P-LW, Y-RY, and S-FH interpreted the data and prepared the manuscript. All authors approved the final manuscript for submission.

## Conflict of Interest

The authors declare that the research was conducted in the absence of any commercial or financial relationships that could be construed as a potential conflict of interest.

## Publisher’s Note

All claims expressed in this article are solely those of the authors and do not necessarily represent those of their affiliated organizations, or those of the publisher, the editors and the reviewers. Any product that may be evaluated in this article, or claim that may be made by its manufacturer, is not guaranteed or endorsed by the publisher.

## Abbreviations

BG: basal ganglia
CV: coefficients of variation
DLPFC: dorsal lateral prefrontal cortex
DTC: dual-task cost
EMG: electromyography
M1: primary motor cortex
MEP: motor evoked potential
MMSE: Mini-Mental State Examination
PD: Parkinson’s disease
RMT: resting motor threshold
SP: silent period
TA: tibialis anterior
tDCS: transcranial direct current stimulation
TMS: transcranial magnetic stimulation
TUG: timed up and go test
UPDRS: Unified Parkinson’s Disease Rating Scale.

## References

[B1] Al-Yahya E. Mahmoud W. Meester D. Esser P. Dawes H. (2019). Neural substrates of cognitive motor interference during walking; Peripheral and Central Mechanisms. *Front. Hum. Neurosci.* 12:536. 10.3389/fnhum.2018.00536 30687049 PMC6333849

[B2] Benecke R. Rothwell J. C. Dick J. P. Day B. L. Marsden C. D. (1986). Performance of simultaneous movements in patients with Parkinson’s disease. *Brain* 109 739–757. 10.1093/brain/109.4.739 3730813

[B3] Beurskens R. Helmich I. Rein R. Bock O. (2014). Age-related changes in prefrontal activity during walking in dual-task situations: a fNIRS study. *Int. J. Psychophysiol.* 92 122–128. 10.1016/j.ijpsycho.2014.03.005 24681355

[B4] Bunai T. Hirosawa T. Kikuchi M. Fukai M. Yokokura M. Ito S. (2021). tDCS-induced modulation of GABA concentration and dopamine release in the human brain: a combination study of magnetic resonance spectroscopy and positron emission tomography. *Brain Stimul.* 14 154–160. 10.1016/j.brs.2020.12.010 33359603

[B5] Collette F. Olivier L. Van der Linden M. Laureys S. Delfiore G. Luxen A. (2005). Involvement of both prefrontal and inferior parietal cortex in dual-task performance. *Brain Res Cogn Brain Res.* 24 237–51. 10.1016/j.cogbrainres.2005.01.023 15993762

[B6] Dedoncker J. Brunoni A. R. Baeken C. Vanderhasselt M. A. (2016). A Systematic Review and Meta-Analysis of the Effects of Transcranial Direct Current Stimulation (tDCS) Over the Dorsolateral Prefrontal Cortex in Healthy and Neuropsychiatric Samples: influence of Stimulation Parameters. *Brain Stimul.* 9 501–517. 10.1016/j.brs.2016.04.006 27160468

[B7] Ferrucci R. Cortese F. Bianchi M. Pittera D. Turrone R. Bocci T. (2016). Cerebellar and motor cortical transcranial stimulation decrease levodopa-induced dyskinesias in Parkinson’s disease. *Cerebellum* 15 43–47. 10.1007/s12311-015-0737-x 26542731

[B8] Ferrucci R. Cortese F. Priori A. (2015). Cerebellar tDCS: how to do it. *Cerebellum* 14 27–30. 10.1007/s12311-014-0599-7 25231432 PMC4318979

[B9] Fisher B. E. Wu A. D. Salem G. J. Song J. Lin C. H. Yip J. (2008). The effect of exercise training in improving motor performance and corticomotor excitability in people with early Parkinson’s disease. *Arch. Phys. Med. Rehabil.* 89 1221–1229. 10.1016/j.apmr.2008.01.013 18534554 PMC2989816

[B10] Fonteneau C. Redoute J. Haesebaert F. Le Bars D. Costes N. Suaud-Chagny M. F. (2018). Frontal transcranial direct current stimulation induces dopamine release in the ventral striatum in human. *Cereb. Cortex.* 28 2636–2646. 10.1093/cercor/bhy093 29688276 PMC5998959

[B11] Fregni F. Boggio P. S. Santos M. C. Lima M. Vieira A. L. Rigonatti S. P. (2006). Noninvasive cortical stimulation with transcranial direct current stimulation in Parkinson’s disease. *Mov. Disord.* 21 1693–1702. 10.1002/mds.21012 16817194

[B12] Fukai M. Bunai T. Hirosawa T. Kikuchi M. Ito S. Minabe Y. (2019). Endogenous dopamine release under transcranial direct-current stimulation governs enhanced attention: a study with positron emission tomography. *Transl Psychiatry.* 9:115. 10.1038/s41398-019-0443-4 30877269 PMC6420561

[B13] Gaspar P. Duyckaerts C. Alvarez C. Javoy-Agid F. Berger B. (1991). Alterations of dopaminergic and noradrenergic innervations in motor cortex in Parkinson’s disease. *Ann. Neurol.* 30 365–374. 10.1002/ana.410300308 1683212

[B14] Groppa S. Oliviero A. Eisen A. Quartarone A. Cohen L. G. Mall V. (2012). A practical guide to diagnostic transcranial magnetic stimulation: report of an IFCN committee. *Clin. Neurophysiol.* 123 858–882. 10.1016/j.clinph.2012.01.010 22349304 PMC4890546

[B15] Hausdorff J. M. Cudkowicz M. E. Firtion R. Wei J. Y. Goldberger A. L. (1998). Gait variability and basal ganglia disorders: stride-to-stride variations of gait cycle timing in Parkinson’s disease and Huntington’s disease. *Mov. Disord.* 13 428–437. 10.1002/mds.870130310 9613733

[B16] Ilg W. Timmann D. (2013). Gait ataxia–specific cerebellar influences and their rehabilitation. *Mov. Disord.* 15 1566–1575. 10.1002/mds.25558 24132845

[B17] Jayaram G. Tang B. Pallegadda R. Vasudevan E. V. Celnik P. Bastian A. (2012). Modulating locomotor adaptation with cerebellar stimulation. *J. Neurophysiol.* 107 2950–2957. 10.1152/jn.00645.2011 22378177 PMC3378372

[B18] Kapogiannis D. Wassermann E. M. (2008). Transcranial magnetic stimulation in Clinical Pharmacology. *Cent. Nerv. Syst. Agents Med. Chem.* 8 234–240. 10.2174/187152408786848076 19122782 PMC2613312

[B19] Kaski D. Dominguez R. O. Allum J. H. Islam A. F. Bronstein A. M. (2014). Combining physical training with transcranial direct current stimulation to improve gait in Parkinson’s disease: a pilot randomized controlled study. *Clin. Rehabil.* 28 1115–1124. 10.1177/0269215514534277 24849794

[B20] Kelly V. E. Eusterbrock A. J. Shumway-Cook A. (2012). A review of dual-task walking deficits in people with Parkinson’s disease: motor and cognitive contributions, mechanisms, and clinical implications. *Parkinsons Dis.* 2012:918719. 10.1155/2012/918719 22135764 PMC3205740

[B21] Kishore A. Meunier S. Popa T. (2014). Cerebellar influence on motor cortex plasticity: behavioral implications for Parkinson’s disease. *Front. Neurol.* 5:68. 10.3389/fneur.2014.00068 24834063 PMC4018542

[B22] Lattari E. Costa S. S. Campos C. de Oliveira A. J. Machado S. Maranhao Neto G. A. (2017). Can transcranial direct current stimulation on the dorsolateral prefrontal cortex improves balance and functional mobility in Parkinson’s disease? *Neurosci Lett.* 636 165–169. 10.1016/j.neulet.2016.11.019 27838447

[B23] Lees A. J. Hardy J. Revesz T. (2009). Parkinson’s disease. *Lancet* 373 2055–2066. 10.1016/S0140-6736(09)60492-X 19524782

[B24] Lefaucheur J. P. (2005). Motor cortex dysfunction revealed by cortical excitability studies in Parkinson’s disease: influence of antiparkinsonian treatment and cortical stimulation. *Clin. Neurophysiol.* 116 244–253. 10.1016/j.clinph.2004.11.017 15661100

[B25] Liu X. Liu H. Liu Z. Rao J. Wang J. Wang P. (2021). Transcranial Direct Current Stimulation for Parkinson’s Disease: a Systematic Review and Meta-Analysis. *Front. Aging Neurosci.* 28:746797. 10.3389/fnagi.2021.746797 34776931 PMC8584149

[B26] Lu C. F. Liu Y. C. Yang Y. R. Wu Y. T. Wang R. Y. (2015). Maintaining Gait Performance by Cortical Activation during Dual-Task Interference: a Functional Near-Infrared Spectroscopy Study. *PLoS One* 10:e0129390. 10.1371/journal.pone.0129390 26079605 PMC4469417

[B27] Manenti R. Brambilla M. Rosini S. Orizio I. Ferrari C. Borroni B. (2014). Time up and go task performance improves after transcranial direct current stimulation in patient affected by Parkinson’s disease. *Neurosci Lett.* 580 74–77. 10.1016/j.neulet.2014.07.052 25107738

[B28] Mishra R. K. Thrasher A. T. (2021). Transcranial direct current stimulation of dorsolateral prefrontal cortex improves dual-task gait performance in patients with Parkinson’s disease: a double blind, sham-controlled study. *Gait Posture* 84 11–16. 10.1016/j.gaitpost.2020.11.012 33260076

[B29] Morris S. Morris M. E. Iansek R. (2001). Reliability of measurements obtained with the Timed “Up & Go” test in people with Parkinson disease. *Phys. Ther.* 81 810–818. 10.1093/ptj/81.2.810 11175678

[B30] Nakashima K. Wang Y. Shimoda M. Sakuma K. Takahashi K. (1995). Shortened silent period produced by magnetic cortical stimulation in patients with Parkinson’s disease. *J. Neurol. Sci.* 130 209–214. 10.1016/0022-510x(95)00029-2 8586988

[B31] Nieuwhof F. Reelick M. F. Maidan I. Mirelman A. Hausdorff J. M. Olde Rikkert M. G. (2016). Measuring prefrontal cortical activity during dual task walking in patients with Parkinson’s disease: feasibility of using a new portable fNIRS device. *Pilot Feasibility Stud.* 2:59. 10.1186/s40814-016-0099-2 27965875 PMC5154104

[B32] Paulus W. Classen J. Cohen L. G. Large C. H. Di Lazzaro V. Nitsche M. (2008). State of the art: pharmacologic effects on cortical excitability measures tested by transcranial magnetic stimulation. *Brain Stimul.* 1 151–163. 10.1016/j.brs.2008.06.002 20633382

[B33] Polanía R. Paulus W. Nitsche M. A. (2012). Modulating cortico-striatal and thalamo-cortical functional connectivity with transcranial direct current stimulation. *Hum. Brain Mapp.* 33 2499–2508. 10.1002/hbm.21380 21922602 PMC6870027

[B34] Potvin-Desrochers A. Paquette C. (2021). Potential Non-invasive Brain Stimulation Targets to Alleviate Freezing of Gait in Parkinson’s Disease. *Neuroscience* 468 366–376. 10.1016/j.neuroscience.2021.05.037 34102265

[B35] Purves D. Augustine G. J. Fitzpatrick D. Hall W. C. LaMantia A. S. McNamara J. O. (2001). *Neuroscience. second ed.* United States: Sinauer Associates.

[B36] Raffegeau T. E. Krehbiel L. M. Kang N. Thijs F. J. Altmann L. J. P. Cauraugh J. H. (2019). A meta-analysis: Parkinson’s disease and dual-task walking. *Parkinson. Relat. Disord.* 62 28–35. 10.1016/j.parkreldis.2018.12.012 30594454 PMC8487457

[B37] Rossini P. M. Burke D. Chen R. Cohen L. G. Daskalakis Z. Di Iorio R. (2015). Non-invasive electrical and magnetic stimulation of the brain, spinal cord, roots and peripheral nerves: basic principles and procedures for routine clinical and research application. An updated report from an I.F.C.N. Committee. *Clin. Neurophysiol.* 126 1071–1107. 10.1016/j.clinph.2015.02.001 25797650 PMC6350257

[B38] Sánchez-Kuhn A. Pérez-Fernández C. Cánovas R. Flores P. Sánchez-Santed F. (2017). Transcranial direct current stimulation as a motor neurorehabilitation tool: an empirical review. *Biomed. Eng. Online* 16:76. 10.1186/s12938-017-0361-8 28830433 PMC5568608

[B39] Schabrun S. M. Lamont R. M. Brauer S. G. (2016). Transcranial direct current stimulation to enhance dual-task gait training in Parkinson’s disease: a pilot RCT. *PLoS One* 11:e0158497. 10.1371/journal.pone.0158497 27359338 PMC4928827

[B40] Shine J. M. Matar E. Ward P. B. Frank M. J. Moustafa A. A. Pearson M. (2013). Freezing of gait in Parkinson’s disease is associated with functional decoupling between the cognitive control network and the basal ganglia. *Brain* 136 3671–3681. 10.1093/brain/awt272 24142148

[B41] Strouwen C. Molenaar E. A. Münks L. Keus S. H. Bloem B. R. Rochester L. (2015). Dual tasking in Parkinson’s disease: should we train hazardous behavior? *Expert Rev. Neurother.* 15 1031–1039. 10.1586/14737175.2015.1077116 26289490

[B42] Verheyden G. Purdey J. Burnett M. Cole J. Ashburn A. (2013). Immediate effect of transcranial direct current stimulation on postural stability and functional mobility in Parkinson’s disease. *Mov. Disord.* 28 2040–2041. 10.1002/mds.25640 24038520

[B43] Werhahn K. J. Kunesch E. Noachtar S. Benecke R. Classen J. (1999). Differential effects on motorcortical inhibition induced by blockade of GABA uptake in humans. *J. Physiol.* 517 591–597. 10.1111/j.1469-7793.1999.0591t.x 10332104 PMC2269337

[B44] Workman C. D. Fietsam A. C. Uc E. Y. Rudroff T. (2020). Cerebellar transcranial direct current stimulation in people with Parkinson’s disease: a pilot study. *Brain Sci.* 10 96. 10.3390/brainsci10020096 32053889 PMC7071613

[B45] Wu A. D. Petzinger G. M. Lin C. H. Kung M. Fisher B. (2007). Asymmetric corticomotor excitability correlations in early Parkinson’s disease. *Mov Disord.* 22 1587–1593. 10.1002/mds.21565 17523196

[B46] Wu T. Hallett M. (2005). A functional MRI study of automatic movements in patients with Parkinson’s disease. *Brain* 128 2250–2259. 10.1093/brain/awh569 15958505

[B47] Yang Y. R. Cheng S. J. Lee Y. J. Liu Y. C. Wang R. Y. (2019). Cognitive and motor dual task gait training exerted specific training effects on dual task gait performance in individuals with Parkinson’s disease: a randomized controlled pilot study. *PLoS One* 14:e0218180. 10.1371/journal.pone.0218180 31220121 PMC6586283

[B48] Yang Y. R. Tseng C. Y. Chiou S. Y. Liao K. K. Cheng S. J. Lai K. L. (2013). Combination of rTMS and treadmill training modulates corticomotor inhibition and improves walking in Parkinson disease: a randomized trial. *Neurorehabil. Neural. Repair.* 27 79–86. 22785003 10.1177/1545968312451915

[B49] Yuan P. Raz N. (2014). Prefrontal cortex and executive functions in healthy adults: a meta-analysis of structural neuroimaging studies. *Neurosci. Biobehav. Rev.* 42 180–192. 10.1016/j.neubiorev.2014.02.005 24568942 PMC4011981

[B50] Zhou J. Manor B. Yu W. Lo O. Y. Gouskova N. Salvador R. (2021). Targeted tDCS Mitigates Dual-Task Costs to Gait and Balance in Older Adults. *Ann. Neurol.* 90 428–439. 10.1002/ana.26156 34216034 PMC8434977

